# The Role of Different Bio-additives in the Properties οf Cement Mortars

**DOI:** 10.1007/s43615-025-00506-6

**Published:** 2025-02-06

**Authors:** P. Kampragkou, M. Stefanidou

**Affiliations:** Laboratory of Building Materials, School of Civil Engineering, Aristotle University of Thessaloniki, Thessaloniki, Greece

**Keywords:** Cement Mortars, Bio-additives, Bio-powders, Sustainability

## Abstract

Nowadays, the research in the field of building materials revolves around the basic principles of sustainability, environmental protection, circular economy and proper management of natural and financial resources. In this context, the current study aimed to implement several biomaterials of diverse species and geometry into cement mortar mixtures and to evaluate their physical, mechanical and hydrothermal properties. Thus, black pine wood and bark powder, pellet crumb and agro-pasture and hemp fibers were incorporated at 1.5% v/v addition rate in cement mortars. After 28 hardening days, all bio-enhanced mortar samples presented advanced flexural strength and decreased specific gravity and thermal conductivity records in relation to the reference-unreinforced case. Furthermore, the powder-crumb like biomaterials seemed to perform as fillers reducing the porosity and capillary absorption values of the specimens compared to the reference composition. From the analysis it seems that biomaterials could provide low cost and eco-friendly reinforcement solutions in the construction sector.

## Introduction

Nowadays, in the context of enhancing the viability of the construction sector, green building proposals are constantly being sought. The promotion of this innovation aims to reduce the environmental degradation and the overconsumption of mineral resources, while minimizing the energy consumption and the CO_2_ emissions from the production of new building materials. Furthermore, in the context of improving the edifices’ eco-profile, efforts are made for the enhancement of the buildings’ energy efficiency by reducing the thermal conductivity performance of the building materials. The specific objective could be achieved through the utilization of low carbon footprint bio- materials [1, 2].

Τhe practice of using bio-products in structural applications dates to ancient times. Historically, natural fibers such as wood, straw and animal hair were employed in traditional mortars to improve volume stability, reduce cracking and increase the strength behavior of the final product. After a period of modernization and replacement of the renewable and biodegradable bio-fibers with metallic and plastic ones, the re-emergence of bio-proposals in the construction industry intends on returning to eco-friendly building solutions [3, 4]. The shift to low environmental impact compositions complies with the circular economy principles. This occurs due to the reuse of abundant, low-cost and fossil fuel independent bio-wastes, from forest, fishery and agricultural industries, as materials’ reinforcement [1, 5].

Regarding the bio-reinforced cement mortars performance, an improvement in their volume deformation, flexural strength and fracture energy behavior has been observed when coconut, wood, jute and kelp fibers are utilized [3, 6, 7]. Furthermore, the bio-additives seem to ameliorate the thermal and breathability properties of the cement mortars, which are essential values for providing healthy indoor quality and thermal comfort to the buildings’ residents [6–8]. Nevertheless, the incorporation of natural fibers into mortar mixtures contributes to voids formation and leads to reduced compressive strength, minimizing the opportunities for construction applications [3, 8].

Τhe characteristics of the reinforced compositions are affected by the chemical composition, the geometry and the surface characteristics of the bio-additives. More specifically, their shape and length, in combination with their dispersion, direction and percentage through the mixture, define the behavior of the final product [3, 9]. In this context, many studies have been conducted for the investigation of bio-fibers implementation in cement mortars [2, 3, 6–8]. Although, the knowledge for the bio-powders (such as mussel shell, lavender and black pine tree powder) performance as additives in mortars is limited [10, 11], as the research so far aims at the utilization of bio powders (such as tree bark, seashell waste and eggshell powder) as cement replacement in pastes and mortars [1, 5, 12, 13].

Taking into account the data from this brief literature review, the purpose of the current study was to incorporate bio-materials with different chemical composition (different species) and geometry (fibers, powders and crumb) into cement mortars and evaluate their physical (absorption, open porosity and apparent specific gravity), mechanical (flexural and compressive strength and dynamic modulus of elasticity) and hygrothermal (capillary absorption and thermal conductivity) properties. So, preliminary data for the performance of bio-powders and grits (compared to fiber like cases) as additives in cement mortars mixtures would be presented.

## Materials and Methods

**Fig. 1:**
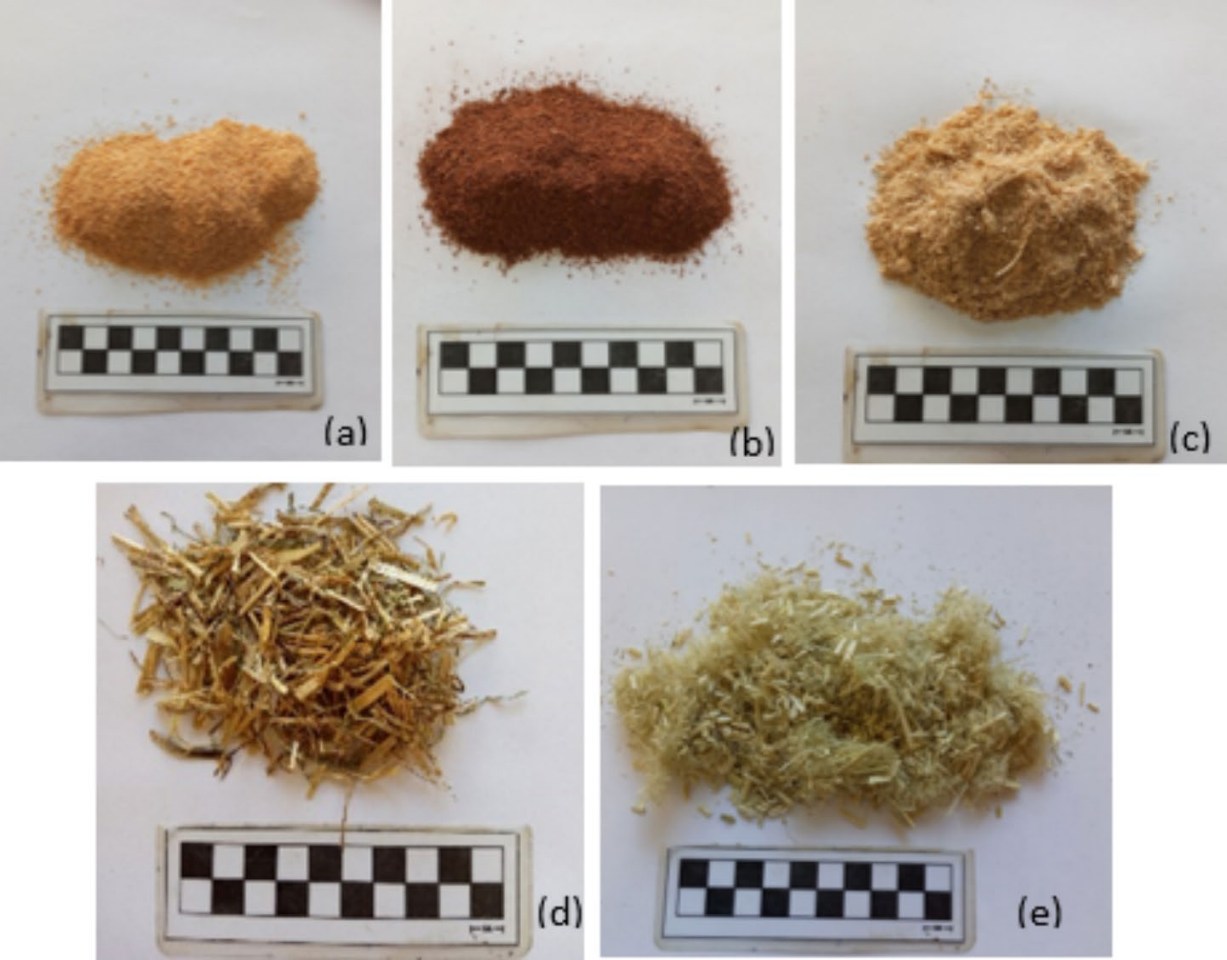
Utilized bio-materials (**a**) black pine wood powder (BG), (**b**) black pine bark powder (BB), (**c**) pellet crumb (P), (**d**) agro-pasture fibers (A) and (**e**) hemp fibers (H)

. For the execution of the experimental procedure, 6 different cement mortar compositions were produced including a reference-unreinforced one (C) and five enhanced with biomaterials of diverse geometry and type. The bio-additives utilized in this research (Fig. 1) were black pine wood powder (0–1 mm diameter) (BG), black pine bark powder (0–1 mm diameter) (BB), pellet crumb (combination of powder and particles 0–1.5 cm length) (P), agro-pasture fibers (1–2 cm length) (A) and hemp fibers (0–2 cm length) (H).

The black pine wood and bark powders were derived as wood processing wastes from industries located in Kalampaka region (Central Greece). Utilizing a hammer mill and a Wiley mill, the wood and bark materials (respectively) were crumbled to powder form and remained for 30 days at dry conditions (20 ± 2 ^o^C and 60 ± 5% relative humidity) until constant mass was achieved. Furthermore, the pellet crumb was obtained from a heating pellet industry in Karditsa (Central Greece), which employes this crumb in the pellet production, while the agro-pasture fibers were natural dried fibers from green lands in North Greece manually cut in 1–2 cm length. Finally, the hemp fibers were obtained as a medical cannabis production residue from a medical industry in Greece. The hemp waste was cut and crushed mechanically with a hammer mill, after the removal of the outside bark layer. The produced hemp material consisted of wood and pith particles.

At each composition the bio-additives were inserted in proportion 1.5% v/v (volume of biomaterial per volume of mortar). More precisely, all the biomaterials were mixed with the cement binder, at a dry state, before the start of the mixing process. The specific implementation rate was defined based on prior study of ancient mortars, where the incorporated biomaterial amount was often not transcending this value [14]. Furthermore, it should be mentioned that different portions of superplasticizer (% w/w: weight of superplasticizer per weight of binder) were added gradually during the mixing procedure (together with water), seeking to preserve the water/binder ratio stable (w/b), while achieving the expected workability (11 ± 1 cm) according to EN 1015-3.Finally, the produced cement mixtures were placed inside pre-oiled molds in multiple layers and externally vidrated with a concrete vibrating table. The duration for the whole mixting procedure lasted approximately 4 min.

The analytic composition of the produced cement mortars together with their workability are displayed in Table 1. Moreover, concerning the samples curing conditions, the cement compositions remained inside the molds in laboratory conditions (20–25^o^C temperature and 50 ± 5% relative humidity) for 24 h and then, after their demolding, the specimens were maintained in a climatic chamber with 20^o^C and 95% relative humidity until the age of 28 days. Finally, it should be noticed that for the execution of the measurements prismatic specimens and slabs were produced with dimensions 4*4*16 cm^3^ and 20*20*2.5 cm^3^ respectively. From Table 1 it is obvious that the hemp fibers are the most absorbent material as they require the largest portion of superplasticizer.

**Table 1:** Composition of the produced mortars (in proportion by weight)

Mortars	Cement I 42.5	Natural river sand (0–4 mm)	Bio-additives (% v/v)	w/c	Superplasticizer (% w/w)	Workability (cm)
C	1	2.5	-	0.45	-	11.0
BG	1	2.5	1.5	0.45	1.0	11.1
P	1	2.5	1.5	0.45	1.5	11.6
A	1	2.5	1.5	0.45	1.5	11.0
BB	1	2.5	1.5	0.45	1.0	11.2
H	1	2.5	1.5	0.45	2.0	10.8

The determination of the mortars’ physical characteristics (absorption, open porosity and apparent specific gravity) was based on RILEM CPC11.3 and conducted at three half prisms for each composition. Furthermore, their mechanical characteristics (flexural and compressive strength) were designated according to EN 1015-11 at three prismatic specimens and 6 half prisms (remaining parts from the flexural test) respectively, utilizing a hydraulic universal strength testing machine with 300kN max testing load and 0.001 mm displacement resolution (loading rate 0.1 kN/s and 1 kN/s for the flexural and compressive strength test respectively). Moreover, the dynamic modulus of elasticity was defined in accordance with EN 12504-4, while the ultrasonic pulse velocity of 3 prismatic specimens for each composition was determined with the Proceq UPV instrument (Pundit Lab +). As for the moisture properties, the capillary absorption was defined based on EN 1015-18 (at 1 prismatic specimen). Finally, the thermal conductivity measurements were conducted with the commercial instrument HFM 100 Series (EN 12667) applying the heat flow meter apparatus at two different mean heating temperatures (10^ο^C and 20^o^C) at 3 flat-slab specimens for each case. Before the thermal tests the slabs were smoothed to achieve flat and parallel surfaces (confirming with the HFM’s instructions) and remained at a climatic chamber with 20^o^C and 60% relative humidity until constant mass was observed (consecutive weightings).

## Results and Discussion

### Physical Properties

From the evaluation of the experimental results (Table 2), it seemed that the addition of bio additives in cement mortars made the final product lighter. In addition, in terms of the absorption and porosity the presence of the black pine wood and bark powder reduced these properties, in contrast to the other fiber like cases. The addition of black pine wood powder (BG) contributed to the highest porosity reduction (about 6%) and the implementation of agro-pasture fibers (A) led to the highest porosity increase (about 114%) compared to the reference mortars. In case of the pellet crumb (P), the specific characteristics were similar with the reference composition. This phenomenon could be attributed to the geometry of the bio-materials, where in the powder like cases their geometry probably improved the stirring procedure during the mortars’ production resulting in a uniform distribution without clumps at the final products, as opposed to fibers’ action. Thus, the powder bio-products prevented the production of further voids, while they defrayed existing ones.

**Table 2:** Values of the absorption, porosity and specific gravity of the produced mortars

Mortars	Absorption (%)	Open porosity (%)	Apparent specific gravity (g/cm^3^)
C	2.75 ± 0.23	6.03 ± 0.49	2.19 ± 0.15
BG	2.71 ± 0.10	5.65 ± 0.38	2.09 ± 0.13
P	3.02 ± 0.21	6.39 ± 0.51	2.12 ± 0.18
A	6.32 ± 0.55	12.90 ± 0.88	2.04 ± 0.21
BB	2.72 ± 0.18	5.72 ± 0.26	2.10 ± 0.17
H	4.79 ± 0.40	9.77 ± 0.75	2.04 ± 0.14

### Mechanical Characteristics

**Fig. 2:**
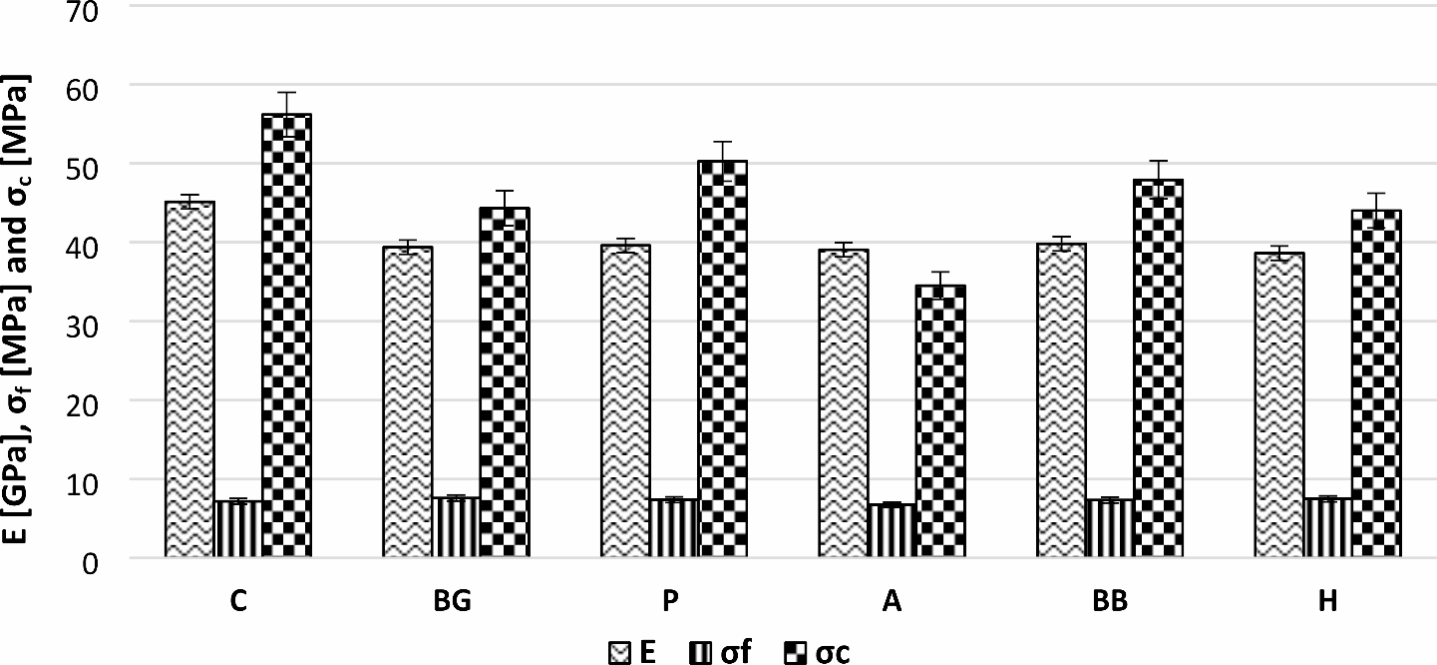
Dynamic modulus of elasticity (E), flexural strength (σ_f_) and compressive strength (σ_c_) of the produced mortars

According to the mechanical properties (Fig. 2), the reinforced mortars (except the P: agro-pasture fiber reinforced samples) presented advanced flexural strength (7.36–7.61 MPa) and declined compressive one (44.03–50.27 MPa), compared to the reference mortars (7.19 MPa and 56.20 MPa respectively). This behavior corresponds with the literature [3, 8, 11], where the incorporation of bio-materials (like wood, diss and doum fibers or black pine wood powder) into cement mortar mixtures induces an improvement in the flexural performance of the cement samples by crack bridging and a decrease in the compressive behavior due to the destruction of hydrogen bonds in the biomaterials-matrix interface. Furthermore, the compressive values comply with the dynamic modulus of elasticity records, which showed that the addition of bio-materials affected negatively the mortars’ rigidity. The best flexural performance showed the black pine wood powder enhanced specimens (BG) (about 6% improvement) and the pellet reinforced (P) presented the lowest compressive reduction of about 11%. As for the A series mortars, they displayed a reduction in both strength properties, which can be justified by their high porosity values in comparison with the C case (114% increase). In terms of the geometry action, all compositions displayed comparable flexural strength (except A case) and the powder-crumb cases showed better compressive results than the fiber reinforced mortars, which can be justified by their porosity records. So, it seems that the powder-crumb bio-additives are able to improve the mechanical performance of the cement mortars despite their fine shape - small grain size.

### Hygrothermal Properties

**Fig. 3:**
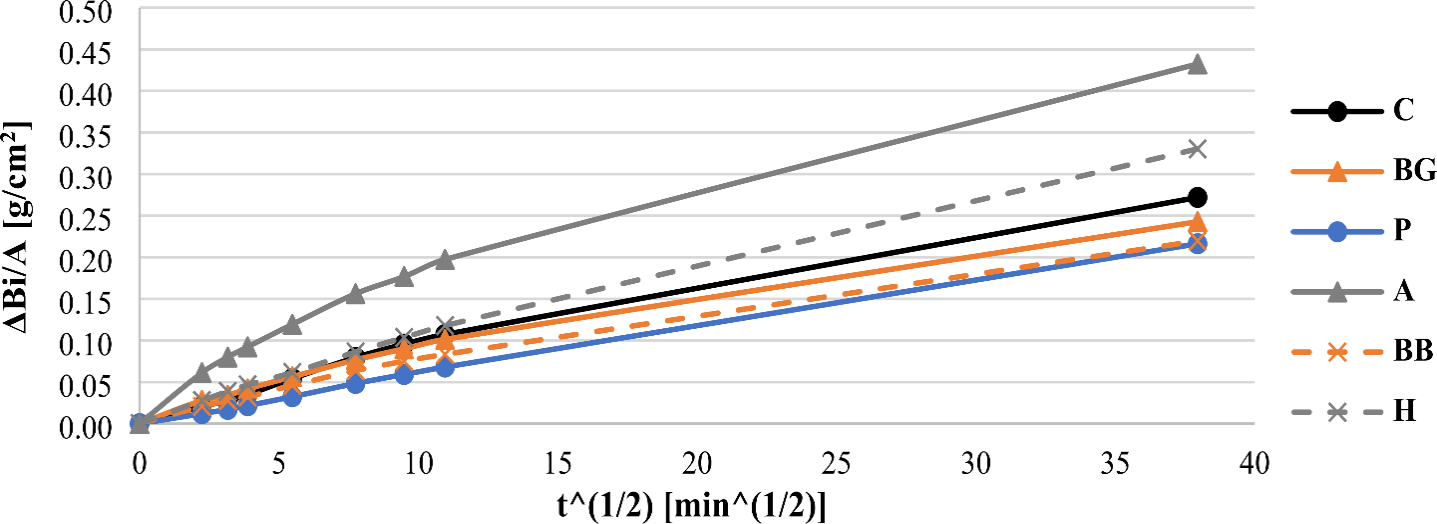
Capillary absorption curves of the cement mortars

Regarding the characterization of the mortars’ moisture performance, capillary absorption tests were conducted. Analyzing the results displayed at Fig. 3, the BG, BB and P compositions presented lower tendency for capillary absorption than the C case, in contrast with the amplified capillary rise of the A and H samples. This could be rationalized by their porosity values. So, the geometry of the biomaterials affected the moisture behavior of the cement mortars leading to the conclusion that the powder additives perform as fillers that block the interconnection of the capillary pores. Furthermore, according to Table 3 the P samples showed the lowest capillary absorption coefficient, about 37% decrease in comparison with the unreinforced composition.

**Table 3:** Capillary water absorption coefficient (C_10−90_) of the produced mortars (measured between the 10 min and 90 min records)

Mortars	C_10−90_ [kg/(m^2^*min^0.5^)]
C	0.11
BG	0.09
P	0.07
A	0.15
BB	0.08
H	0.10

Considering the thermal behavior of the mortars (Table 4), the bio-reinforcements induced a reduction in the thermal conductivity coefficient (λ) of the final products. In the case of the fiber enhanced mortars, which presented higher porosity values than the reference composition, this influence was affected both from the specimens’ porosity records- lighter structure and the thermal conductivity values of the bio-additives themselves. This statement could be explained by the fact that the H composition presented a greater decrease in λ (33% compared to the C composition) and lower porosity value than the A case (20% λ reduction compared to the reference). Although, in case of the powder reinforced compositions, which presented declined porosity records compared to the reference composition, the λ decrease could be justified by the beneficial thermal insulating properties of the bio-materials [15]. Finally, only the P case displayed similar thermal values with the reference, which is in accordance with their similar porosity values.

**Table 4:** Values of the thermal conductivity coefficient (λ) of the produced mortars

Mortars	λ [W/(m*K)]	
	10℃	20℃
C	0.9773 ± 0.0645	0.9856 ± 0.0675
BG	0.8591 ± 0.0458	0.8764 ± 0.0535
P	0.9780 ± 0.0595	1.0051 ± 0.0624
A	0.7836 ± 0.0588	0.7898 ± 0.0486
BB	0.7535 ± 0.0356	0.8412 ± 0.0547
H	0.6568 ± 0.0427	0.6925 ± 0.0562

## Conclusions

To sum up, the aim of the current research was the implementation of biomaterials with diverse chemical composition and geometry into cement mortar mixtures and the evaluation of the final products’ physical, mechanical and hygrothermal properties. For this purpose, black pine wood and bark powder, pellet crumb and agro-pasture and hemp fibers were utilized at 1.5% v/v addition rate. Finally, the assessment of the powder-crumb bio-materials performance in comparison with the fiber like ones could provide preliminary data for the behavior of bio-powders as additives in cement mortars.

Analyzing the experimental results, it seemed that the presence of biomaterials (of all kinds) induced lightness in the cement mortars. Furthermore, the addition of black pine wood and bark powder reduced the porosity and absorption values of the specimens, in contrast with the fiber- like cases, while the pellet crumb did not affect significantly these properties. So, it could be assumed that the bio-powders work as fillers in cement mortar mixtures. The black pine wood powder displayed the best porosity records (−6% deviation from the reference case). As regards the mechanical characteristics of the mortars, the incorporation of biomaterials improved the flexural strength of the samples (except the case with the agro-pasture fibers) but declined the compressive strength. The black pine wood powder had the best flexural performance, and the pellet crumb displayed the lowest compressive reduction. As for the dynamic modulus of elasticity, the bio-additives affected negatively the mortars’ stiffness. In the context of the geometry action, it seemed that it mostly affected the compressive strength with the powder-crumb reinforced cases to perform better than the fiber-like ones, as the flexural results were similar between all cases (except A composition).

Continuing with the hygrothermal characteristics, the powder-crumb reinforced mortars displayed lower tendency for water capillary absorption than the reference mortars, in contrast with the increased capillary rise of the fiber reinforced ones. Furthermore, the presence of biomaterials improved the thermal conductivity performance of the specimens, and this influence was affected by the specimens’ porosity and/ or the bio-additives’ λ value.

Taking all these factors into account, the geometry and morphology of the biomaterials defined the behavior of the final products. The bio-powders seemed to improve the physical, mechanical (flexural strength), moisture and thermal properties of cement mortars leading to the conclusion that these fine bio-particles worked as fillers and could prove to be promising bio-reinforcements. Among the studied biomaterials, the black pine wood and bark powders presented the most preferable results considering the overall mortars behavior.

So, the bio-reinforcements could provide low cost and eco-friendly green construction solutions. Further research on the suitable bio-products addition rate, the microstructure and the post cracking behavior of the final compositions could lead to a better understanding of the role of the biomaterials in the cement mortars’ performance.
